# Supplementary material for: Targeting Integrin α3 Blocks β1 Maturation, Triggers Endoplasmic Reticulum Stress, and Sensitizes Glioblastoma Cells to TRAIL-Mediated Apoptosis
Source: Cells. 2024 Apr 26;13(9):753. doi: 10.3390/cells13090753 (PMC11083687; doi:10.3390/cells13090753)
Supplement: Supplementary file 1 [file cells-13-00753-s001.zip › Suppl Methods and Suppl Figure legends 04252024.pdf]

Supplementary Materials and Methods

Reagents

Short-interfering RNAs:

Transient siRNA knockdown for all integrin subunits and control treatment were carried out using the following validated siRNAs. Silencer Select siRNAs (Thermo Fisher Scientific, Waltham, MA, USA): ITGA3 (#4390824 ID: s7542, and #4427038 ID: s7543; s7542 was mainly used in this study), ITGA1 (#4390824 ID: s7532), ITGA4 (#4390824 ID: s7545), ITGA5 (#4390824 ID: s7547), ITGA7 (#4390824 ID: s7552), and ITGAV (#4390824 ID: s7570). Silencer pre-designed siRNAs (Thermo Fisher Scientific): ITGA9 (#AM16708, ID: 114980). Santa Cruz Biotechnology (SCBT; Dallas, TX, USA) siRNAs: ITGA2 (sc-29371), ITGA6 (sc-43129), ITGA8 (sc-35688), ITGA10 (sc-88849), ITGA11 (sc-90047), ITGB1 (sc-35674), BAX (sc-29212), Talin-1 (sc-36610), FAK (sc-29310), DR5 (sc-40237), and Control siRNA-A (sc-37007). All siRNAs were transfected in the cells at 30 nM concentration.

Antibodies:

Integrin α3 (sc-374242), α4 (sc-14008), α5 (sc-10729), β1 (sc-374429), BAX (sc-23959), caspase 3 (sc-7148), and β-actin (sc-69879) antibodies were purchased from SCBT and the dilution that was used in immunoblot assays was 1:500, except for α3 and β-actin (1:2000). Integrin αv (#4711), PARP (#9542), c-PARP (#5625), phospho-Akt (S473, #4060), Akt (#9272), Talin (#4021), FAK (#13009), caspase 8 (#9746), caspase 9 (#9502), DR5 (#8074), and BiP (#3177) antibodies were purchased from Cell Signaling Technology (CST; Danvers, MA, USA) and diluted to 1:1000 for immunoblot analyses. Goat anti-rabbit or mouse secondary antibodies were purchased from Thermo Fisher Scientific (#31460 and #31430, respectively). For immunoprecipitation assays, 5 µg of mouse IgG (sc-2025, SCBT), rabbit IgG (sc-2027, SCBT),

DR5, and integrin β1 antibodies were used.

Chemicals:

TRAIL (310-04, Peprotech, Cranbury, NJ, USA) used at 100 µg/ml in media. Caspases inhibitors: Caspase-3/7 Inhibitor (218826, Sigma-Aldrich, Burlington, MA, USA), caspase-8 Inhibitor Z-IETD-FMK (sc-3084, SCBT), and pan-caspase inhibitor Z-VAD-FMK (sc-3067, SCBT). ER stress inhibitors: Salubrinal (PERK inhibitor, sc-202332, SCBT) and STF083010 (IRE inhibitor, sc-474562, SCBT)

Plasmids:

Expression vectors for the following proteins were used: wild type integrin α3 and ITGA3-mut 5 (Vectorbuilder, Chicago, IL, USA) contains five silent mutations in the target region for siα3 (ID: s7542). WT ITGA3 (from codon 2772) 5'-ACC AAC GTG ACT GTG AAG GCA-3', mut 5 (2772) 5'-ACA AAT GTG ACC GTG AAA GCC-3'.

Primers:

Human DR5: 5'-CAAGACCCTTGTGCTCGTTGT-3' (forward) and 5'-GACACATTCGATGTCACTCCA-3' (reverse)

Human integrin **α**3 (ITGA3): 5'-GGTACACGATGCAGGTAGGC-3' (forward) and 5'-TTCAAACGGAGCTCCCACAG-3' (reverse)

Human glyceraldehyde-3-phosphate dehydrogenase (GAPDH): 5'-TGAAGGTCGGAGTCAACGGATTTGGT-3' (forward) and 5'-CATGTGGGCCATGAGGTCCACCAC-3' (reverse)

Methods

Cycloheximide (CHX) chase assay

The protein stability of DR5 was determined by treating LN229 cells with cycloheximide (CHX #01810, Sigma-Aldrich, 200 µg/ml final concentration in culture media) 48h post transfection with siRNAs. Total cell extracts were harvested at 0,1,2,3,4 and 6 hours post CHX treatment. Cells were lysed in 2X Laemmli buffer (#1610737, BioRad, Hercules, CA, USA) and boiled at 95 °C for 5 min before SDS-PAGE electrophoresis. Relative DR5 protein levels were determined by densitometry analysis with imageJ software version 1.53k (https://imagej.nih.gov/ij/) and normalized to internal control β-actin levels.

Supplementary Figure legends

Supplementary Figure S1

Silencing of integrin α3 induces cancer cells-specific apoptosis.

LN229 human GBM cells were transfected with indicated siRNAs (30 nM) and cell extracts analyzed by immunoblotting 48h later unless otherwise indicated.

A. Immunoblot analysis of human glioma cell lines LN-319, LN751, LN444, and LN229 treated with sia3 (30 nM) for 72h. Cells were switched to serum-free medium 48h after transfection and harvested 24h later. Note siα3-mediated induction of apoptosis as detected by cleaved PARP.

B. Immunoblot analysis shows siα3 (30 nM; 48h) activates PARP cleavage (*) in several human cancer cell lines. Note induction of cPARP is independent of p53.

C. Immunoblot analysis of LN229 cells grown for 24h +/- serum starvation followed by 48h siα3 or siβ1 transfection (30 nM). Note increased siα3-mediated PARP cleavage under serum starvation.

D. Immunoblot analysis of LN229 cells 48h after transfection with indicated siRNAs (30 nM). Note reduced cleaved PARP (*) upon Bax knockdown.

E. (Left panel) Cell viability of LN229 and BEAS-2B cells 48h and 96h post siRNA transfection (30 nM). Student's t test (***; $p < 0.001$). (Middle panel) Caspase 3/7 Glo assay in normal lung BEAS-2B cells following 48h transfection with indicated siRNAs (30 nM). Pan-caspase inhibitor Z-VAD (20 μM) was added 24h post-transfection. UV treatment (60 mJ/cm^2) was used as a positive control to induce caspase 3/7 activity. (Right panel) Immunoblot of LN229 and normal human lung BEAS-2B cells after 96h siα3 treatment (30 nM).

Supplementary Figure S2

DR5 mediates integrin a3-targeted cell killing.

A. SRB cell survival assay of indicated cell lines treated for 24h with TRAIL (100 ng/mL), translation inhibitor cycloheximide (CHX; 20 μM), or both.

B. Hoechst 33342 staining of LN229 cells transfected with siCtrl, α3, β1, or siα3/β1 (30 nM, 96h) +/- TRAIL treatment (100 ng/mL, added 24h after siRNA transfection).

C. Immunoblot analysis of LN229 cells transfected with siα3 or siCtrl (30 nM) for 40h. TRAIL (100 ng/ml) +/- Cycloheximide (CHX, 20 μM) were added 24h post-transfection. Note that siα3 sensitizes cells to TRAIL-mediated cleavage of Caspases 8 and 9 (*active forms).

D. Immunoblot analysis of human colorectal cancer HCT116 cell line 48h after siα3, siβ1, or siCtrl transfection (30 nM). Note potent activation of cPARP and reduced β1 integrin maturation in siα3 treated cells.

E. Caspase 8 Glo assay in LN229 cells 48h after transfection of indicated siRNAs (30 nM). Note: addition of Caspase 8 inhibitor Z-IETD (20 μM) 4h post-transfection prevented siα3-mediated caspase 8 activation.

F. Immunoblot analysis of LN229 cells transfected with siα3 or siCtrl (30 nM) for 48h. TRAIL (100 ng/ml) was added 24h post-transfection. Cells were treated with caspase inhibitors (20 μM) 1h before transfection.

G. Cycloheximide (CHX) chase assay to measure DR5 stability post siα3 treatment (30 nM; 48h) in LN229 cells. (top panel) Immunoblot analysis with time course following translational block with CHX treatment (100 μg/ml). (bottom panel) Quantification of DR5 degradation kinetics with ImageJ.

H. Immunoblot analysis of LN229 cells transfected with siα3 or siCtrl (30nM) for 96h. TRAIL (100 ng/ml) and kifunensine (5 μM) were added 48h post-transfection.
