# Supplementary figures and images for: Targeting Integrin α3 Blocks β1 Maturation, Triggers Endoplasmic Reticulum Stress, and Sensitizes Glioblastoma Cells to TRAIL-Mediated Apoptosis
Source: Cells. 2024 Apr 26;13(9):753. doi: 10.3390/cells13090753 (PMC11083687; doi:10.3390/cells13090753)

Supplementary Figure S1

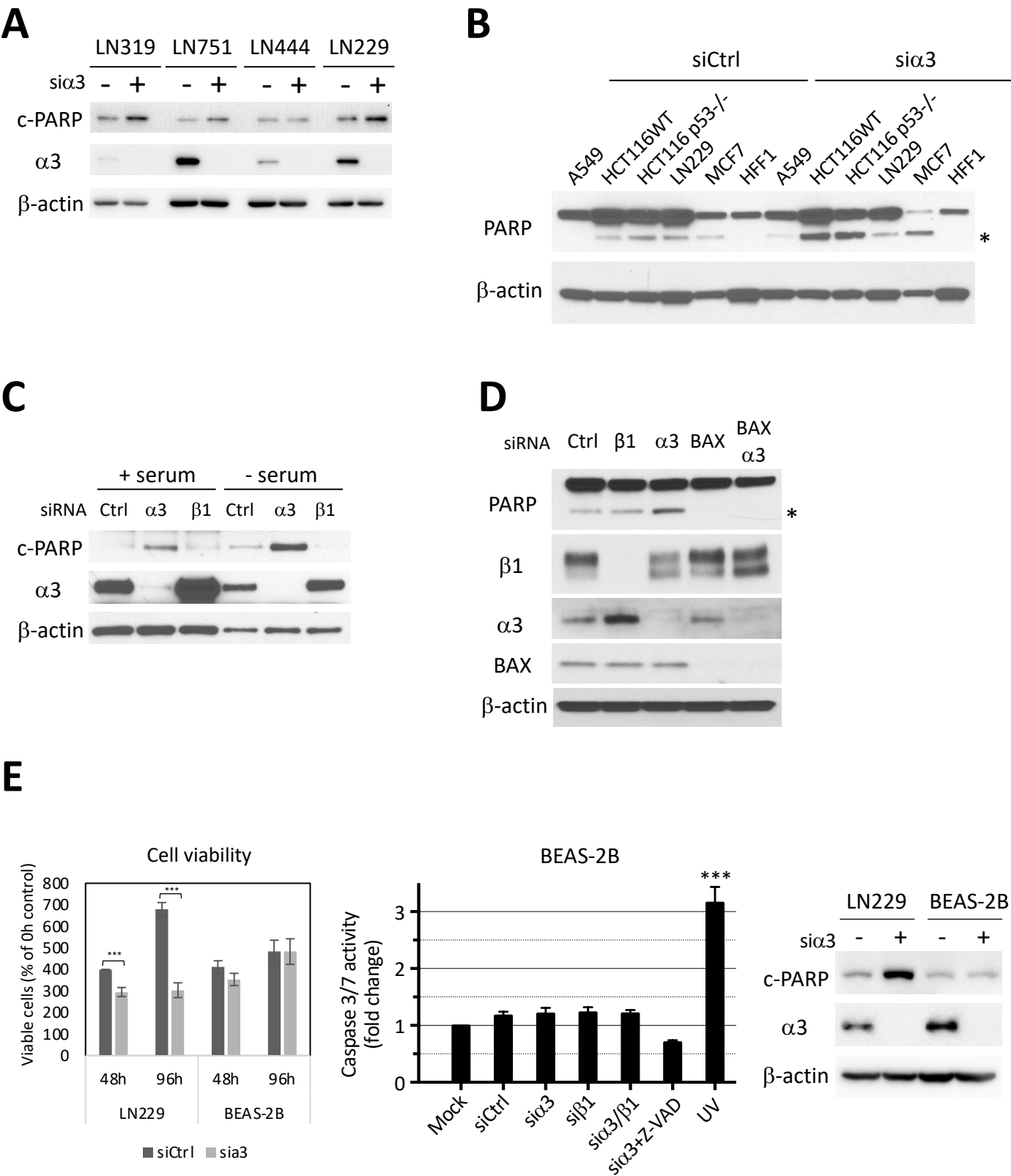

# Supplementary Figure S2

## A

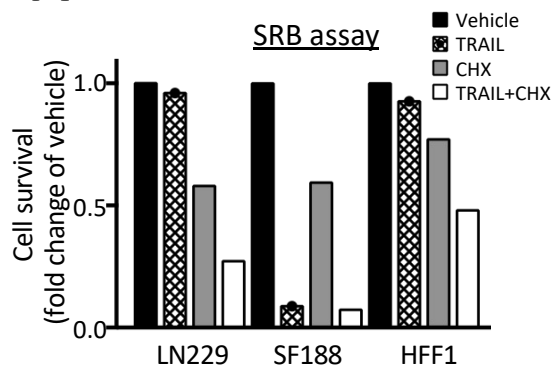

## B

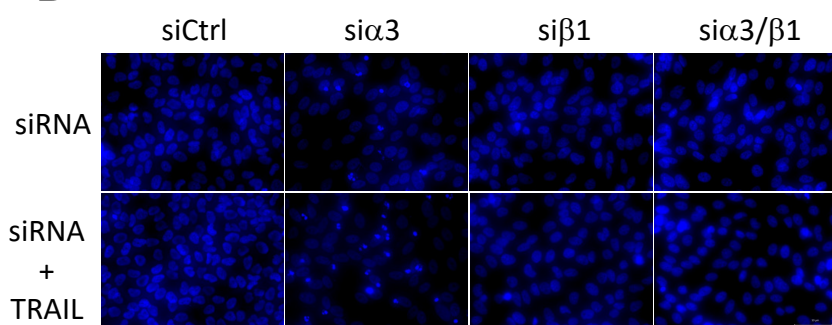

## C

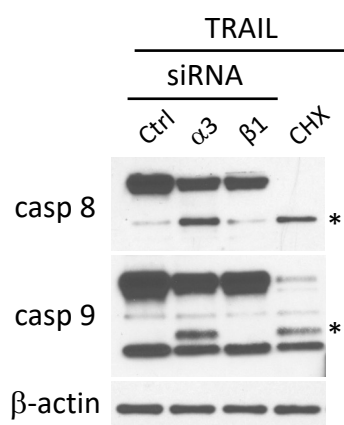

## D

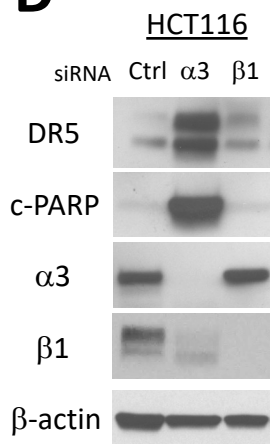

## E

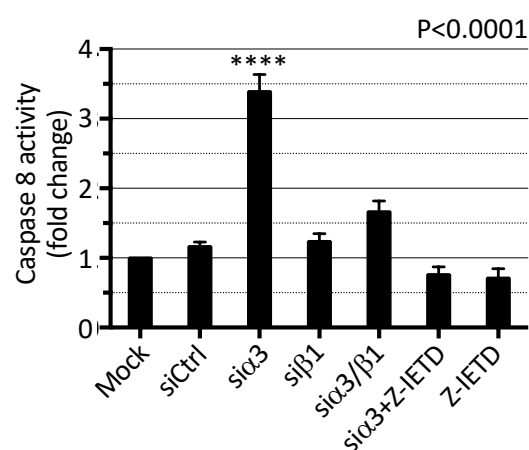

## F

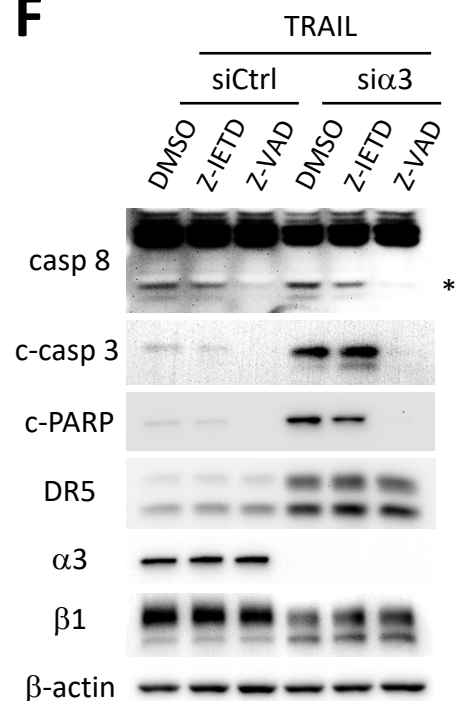

## G

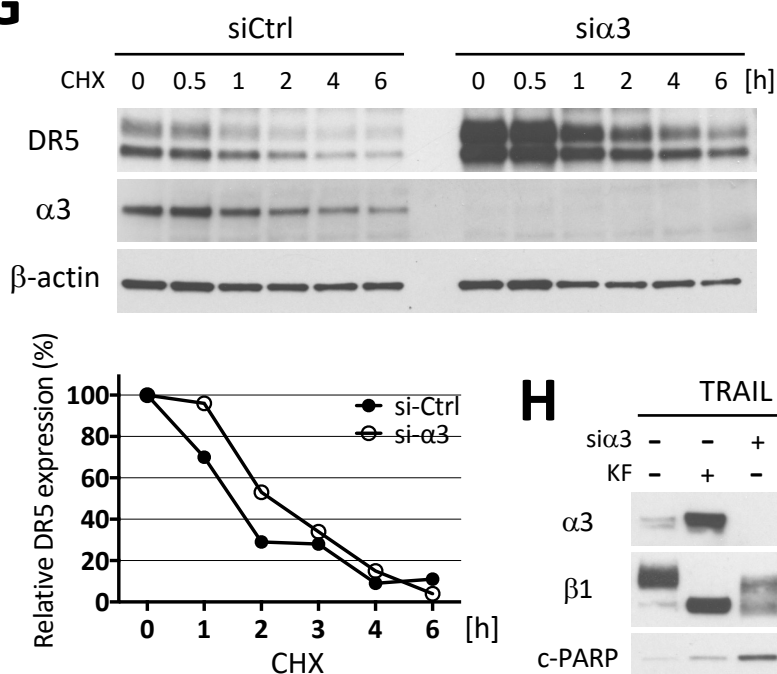

## H

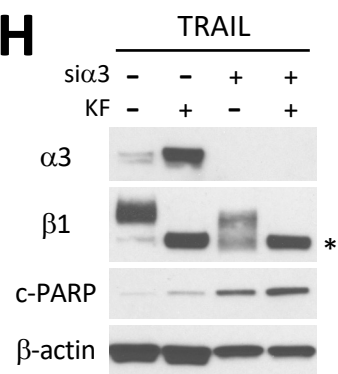

Supplement: Supplementary file 1 [file cells-13-00753-s001.zip › Supplementary figures.pdf]
